# Identifying Predictive Characteristics of Opioid Medication Use among a Nationally Representative Sample of United States Older Adults with Pain and Comorbid Hypertension or Hypercholesterolemia

**DOI:** 10.3390/healthcare8030341

**Published:** 2020-09-15

**Authors:** David R. Axon, Shannon Vaffis, Srujitha Marupuru

**Affiliations:** Department of Pharmacy Practice and Science, University of Arizona College of Pharmacy, Tucson, AZ 85721, USA; vaffis@pharmacy.arizona.edu (S.V.); marupuru@pharmacy.arizona.edu (S.M.)

**Keywords:** opioids, adult, pain management, hypertension, hypercholesterolemia

## Abstract

The prevalence of older adults with pain and comorbid cardiovascular conditions is increasing in the United States (U.S.). This retrospective, cross-sectional database study used 2017 Medical Expenditure Panel Survey data and hierarchical logistic regression models to identify predictive characteristics of opioid use among a nationally representative sample of older U.S. adults (aged ≥50 years) with pain in the past four weeks and comorbid hypertension (pain–hypertension group) or hypercholesterolemia (pain–hypercholesterolemia group). The pain–hypertension group included 2733 subjects (*n* = 803 opioid users) and the pain–hypercholesterolemia group included 2796 subjects (*n* = 795 opioid users). In both groups, predictors of opioid use included: White race versus others, Hispanic versus non-Hispanic ethnicity, 1 versus ≥5 chronic conditions, little/moderate versus quite a bit/extreme pain, good versus fair/poor perceived mental health, functional limitation versus no functional limitation, smoker versus non-smoker, and Northeast versus West census region. In addition, Midwest versus West census region was a predictor in the pain–hypertension group, and 4 versus ≥5 chronic conditions was a predictor in the pain–hypercholesterolemia group. In conclusion, several characteristics of older U.S. adults with pain and comorbid hypertension or hypercholesterolemia were predictive of opioid use. These characteristics could be addressed to optimize individuals’ pain management and help address the opioid overdose epidemic.

## 1. Introduction

Pain is prevalent among United States (U.S.) adults, with estimates ranging from 8.0% to 20.4% in high-impact chronic pain (pain that limited at least one major life activity) and higher prevalence reported among older adults [1]. Among U.S. older adults, back pain is the most commonly reported type of pain, affecting approximately one-third of older adults [2,3]. However, appropriate pain management is often challenging for older adults as it is associated with comorbid conditions, such as cardiovascular conditions [4]. A report from the Global Burden of Disease Study 2016 shows that high prevalence of pain and pain-related diseases is the leading cause of disability and disease burden globally [5]. The number of U.S. adults suffering from at least one non-cancer painful health condition increased substantially from 32.9% in 1997/1998 to 41% in 2013/2014 [6]. 

Although a variety of pain management strategies exist [7], opioids have increasingly been used by older adults to manage both acute and chronic pain in recent years [8]. Commonly prescribed opioids include hydrocodone, oxycodone, oxymorphone, morphine, codeine, and fentanyl [9]. Evidence for the short-term efficacy of opioid use (≤12 weeks) among older adults has been established [10], and opioids have become widely used to manage persistent pain in older adults [11]. Appropriate use of opioids under a clinician’s supervision may provide older adults with necessary pain relief, allowing them to remain active, independent, and able to maintain a higher quality of life [12]. The Centers for Disease Control and Prevention (CDC) guidelines recommend increasing monitoring to minimize the risks of opioids in older adults, yet lack detailed guidance on opioid prescribing [13]. Opioids may be considered when pain has not responded to other strategies or when major functional impairment persists despite treatment [10]. Pain can be difficult to manage in older adults if they are unable to tolerate non-opioid analgesics due to impaired liver or kidney function, hypertension [14], other cardiac risks [15], concomitant anticoagulant therapy in atrial fibrillation or after stroke [16], or risk of gastrointestinal bleeding [17]. 

As a consequence of increased pain prevalence and opioid use, studies have reported an increase in healthcare resource utilization. For example, opioid-related hospitalizations among U.S. adults aged 65 and older increased by 34%, and emergency department visits increased by 74%, between 2010 and 2015 [18]. Recent data have also demonstrated an increase in opioid use disorder hospitalizations among a national inpatient sample to 191 per 100,000 population in 2015–2016 [19]. Correspondingly, a recent study found that among a nationally representative sample of older U.S. adults with pain, opioid users were associated with greater healthcare costs compared to their counterparts who did not use opioids [20].

Most available studies on opioid therapy for non-cancer pain are limited to a short follow-up period of three to six months and are generally not based on real-world data [21]. There is therefore an interest in investigating the predictors of opioid use, particularly among older adults. For example, one study using data from the National Alzheimer’s Coordinating Center found several patient characteristics were associated with higher prevalence of opioid usage among U.S. adults aged 65 years and older [22]. In another instance, a prospective study of patients aged 45 years and older in a large nonprofit healthcare system in Washington State found that expectations about continuing opioid use were significant predictors of long-term (one-year) opioid use [23].

However, the predictors of opioid use among older U.S. adults with pain and chronic conditions such as cardiovascular disease have not been investigated. Cardiovascular conditions, such as hypertension and hypercholesterolemia, are prevalent in the U.S. and were therefore selected as the comorbid conditions for this investigation. Approximately three in five adults over 60 years of age have hypertension, and one in eight adults over 60 years of age has hypercholesterolemia [24,25]. This information will be useful as the number of older adults increases and the opioid public health crisis persists. Therefore, this exploratory study aimed to answer the following research questions: (1) Which characteristics are predictive of opioid medication use among a nationally representative sample of older U.S. adults (≥50 years) with pain and comorbid hypertension? and (2) Which characteristics are predictive of opioid medication use among a nationally representative sample of older U.S. adults (≥50 years) with pain and comorbid hypercholesterolemia? 

## 2. Materials and Methods 

### 2.1. Medical Expenditure Panel Survey (MEPS) Study Design

This study involved Medical Expenditure Panel Survey (MEPS) data from the year 2017 in a retrospective, cross-sectional design. MEPS uses the sampling framework of the previous years’ National Health Interview Survey (NHIS) and a panel design with multiple interview rounds over two calendar years that oversamples disabled and minority groups to obtain a nationally representative sample of non-institutionalized U.S. citizens. The MEPS is conducted on behalf of the Agency for Healthcare Research and Quality (AHRQ) and includes several components. One of these components, the MEPS household component (MEPS-HC), collects self-reported demographic characteristics, health conditions, and prescription medication use for each household member [26].

### 2.2. Eligibility Criteria

Subjects were eligible for inclusion in this study if they were alive for the full year, were aged 50 years or older, had pain in the past four weeks, and had a diagnosis of: (1) hypertension (the pain–hypertension group); or (2) hypercholesterolemia (the pain–hypercholesterolemia group). Subjects who reported “a little bit”, “moderately”, “quite a bit”, or “extremely”, to the item: “During the past 4 weeks, pain interfered with normal work outside the home and housework” were defined as having pain [27,28]. These characteristics were identified from the MEPS 2017 full-year consolidated data file that is publicly available on the MEPS website [29].

### 2.3. Predictor Variables

The predictor variables in this study were conceptualized using Andersen’s Behavioral Model of Health Services Use, which includes the following: (1) predisposing factors; (2) enabling factors; (3) need factors; (4) personal health practices factors; and (5) external environmental factors [30].

The predisposing factors were as follows: age (50–64 years, ≥65 years); ethnicity (Hispanic, non-Hispanic); race (White, other); and sex (male, female).

Enabling factors consisted of education status (less than high school, up to high school, more than high school); employment status (employed, unemployed); marital status (married, other); health insurance provider (private, public, uninsured); and poverty indicator (poor/near poor/low income (<200% federal poverty level), middle/high income (≥200% federal poverty level)).

One need factor was the number of chronic conditions (angina, arthritis, asthma, cancer, chronic bronchitis, coronary heart disease, diabetes, joint pain, emphysema, hypercholesterolemia, hypertension, myocardial infarction, other unspecified heart disease, and stroke) (0, 1, 2, 3, 4, ≥5), although hypertension was not included in the pain–hypertension group and hypercholesterolemia was not included in the hypercholesterolemia–pain group. Other need factors included pain severity (little/moderate, quite a bit/extreme); perceived physical health status (excellent/very good, good, fair/poor); perceived mental health status (excellent/very good, good, fair/poor); activities of daily living (ADL) limitations (defined as needing help with eating, dressing, bathing, toileting, getting in and out of bed, and mobility inside own residence) (yes, no); instrumental activities of daily living (IADL) limitations (defined as needing help or supervision with using the telephone, paying bills, taking medications, preparing light meals, doing laundry, or going shopping) (yes, no); functional limitations (defined as having difficulty lifting ten pounds, walking up ten steps, walking three blocks, walking a mile, standing for 20 min, bending or stooping, reaching overhead, or using fingers to grasp) (yes, no); and work, housework, or school limitations (yes, no). 

The two personal health practices factors were regular exercise (≥30 min moderate to vigorous physical activity at least five times a week) (yes, no) and smoking status (yes, no). 

The only external environmental factor was census region (Northeast, Midwest, South, West) [27,28].

### 2.4. Dependent Variable

The dependent variable in this study was opioid use (opioid user, non-user). Opioid use was indicated if the subject used at least one opioid analgesic or opioid analgesic combination in 2017, which was identified using the Multum Lexicon therapeutic class codes of “60” (narcotic analgesics) or “191” (narcotic analgesic combinations) available in the 2017 prescribed medicines file [29,31,32].

### 2.5. Data Analysis

The two study groups (pain–hypertension group and pain–hypercholesterolemia group) were developed in accordance with the criteria set out above. Chi-square tests were used to determine statistically significant differences between opioid users and non-users. Several hierarchical logistic regression models were developed to identify the factors associated with opioid use. The first logistic regression model included predisposing factors, while subsequent models adjusted for an additional group of factors (enabling, need, personal health practices, and external environmental). Analyses were conducted using SAS University (SAS institute Inc., Cary, NC, USA) and accounted for the complex survey design of MEPS to obtain national estimates. The University of Arizona Institutional Review Board approved this study (1912255773).

## 3. Results

### 3.1. Selection of Study Subjects

A total of 31,880 subjects were available in the 2017 MEPS dataset and were assessed against the eligibility criteria. Accordingly, the pain–hypertension group included 2733 subjects (*n* = 803 opioid users) and the pain–hypercholesterolemia group included 2796 subjects (*n* = 795 opioid users). 

### 3.2. Prevalence of Opioid Medication Use among Study Subjects

The prevalence of opioid use among community-dwelling U.S. adults aged 50 years or older with pain and hypertension was 29.4% (95% confidence interval (CI) = 27.4%, 31.5%) from a weighted population of 29,308,898. Meanwhile, the prevalence of opioid use among those with pain and hypercholesterolemia was 28.5% (95% CI = 26.4%, 30.5%) from a weighted population of 31,014,839. 

### 3.3. Characteristics of Study Subjects

Table 1 reports the characteristics of study subjects. In both the pain–hypertension group and the pain–hypercholesterolemia group, the majority of subjects were aged ≥65 years, non-Hispanic, White, female, educated high school or beyond, unemployed, married, had private health insurance, middle/high income, ≥4 chronic conditions, little/moderate pain, good/very good/excellent physical health, good/very good/excellent mental health, no activities of daily living limitations, no instrumental activities of daily living limitations, no functional limitations, no work, housework, or school limitations, did not regularly exercise, and did not smoke. The most common census region was the South. There were differences between opioid users and non-users for all characteristics (*p* < 0.05) except age (*p* = 0.1279) and health insurance status (*p* = 0.0581) in the pain–hypertension group, and except age (*p* = 0.1246), sex (*p* = 0.6975), education (*p* = 0.0754), and health insurance status (*p* = 0.1626) in the pain–hypercholesterolemia group.

### 3.4. Predictors of Opioid Medication Use

**Figure 1:**
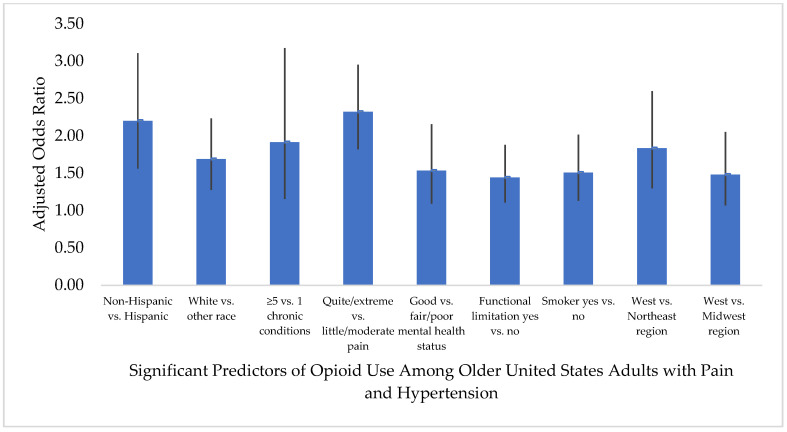
Predictive characteristics of opioid medication use among older United States adults (age ≥50 years) with pain in the past four weeks and a diagnosis of hypertension. Pain–hypertension group: unweighted sample *n* = 2733 (opioid use *n* = 803; no opioid use *n* = 1930) reflects weighted sample *n* = 29,308,898 (opioid users *n* = 8,625,387; non-users *n* = 20,683,511) of United States adults alive during the calendar year 2017, age ≥50 years, with self-reported pain in the past four weeks, and a diagnosis of hypertension. “No opioid use” served as the reference group for the dependent variable in the binomial logistic regression. Only characteristics that were significant predictors of opioid use are included in the figure.

**Figure 2:**
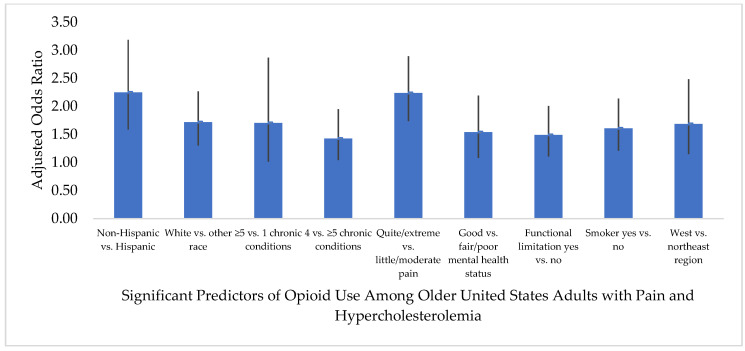
Predictive characteristics of opioid medication use among older United States adults (age ≥50 years) with pain in the past four weeks and a diagnosis of hypercholesterolemia. Pain–hypercholesterolemia group: unweighted sample *n* = 2796 (opioid use *n* = 795; non-users *n* = 2001) reflects weighted sample *n* = 31,014,839 (opioid users *n* = 8,835,304; non-users *n* = 22,179,535) of United States adults alive during the calendar year 2017, age ≥50 years, with self-reported pain in the past four weeks, and a diagnosis of hypercholesterolemia. “No opioid use” served as the reference group for the dependent variable in the binomial logistic regression. Only characteristics that were significant predictors of opioid use are included in the figure.

Figure 1 and Figure 2 report the results of the binomial logistic regression analyses that show the association of predisposing, enabling, need, personal health practices, and external environmental factors with opioid use. In both the pain–hypertension and pain–hypercholesterolemia groups, White race (versus other races) was a predictive characteristic of opioid use; those who were White were approximately 1.7 times more likely to use opioids compared to those of other races (adjusted odds ratio (AOR) = 1.689, 95% CI = 1.278, 2.232 and AOR = 1.717, 95% CI = 1.302, 2.265, respectively). Ethnicity was also a predictor of opioid use in both groups; those who were non-Hispanic were less likely to be opioid users than non-users in the pain–hypertension group (AOR = 0.454, 95% CI = 0.322, 0.640) and pain–hypercholesterolemia group (AOR = 0.445, 95% CI = 0.314, 0.631). 

Need factors were significant predictors of opioid use in both groups; 1 versus ≥5 chronic conditions was a predictor in the pain–hypertension group, (AOR = 0.522, 95% CI = 0.315, 0.866) and in the pain–hypercholesterolemia group (AOR = 0.587, 95% CI = 0.349, 0.986), but 4 versus ≥5 chronic conditions was also a predictor in the pain–hypercholesterolemia group (AOR = 1.425, 95% CI = 1.042, 1.949). Other need factors that were predictors of opioid use included: little/moderate pain severity versus quite a bit/extreme pain severity (pain–hypertension AOR = 0.431, 95% CI = 0.339, 0.549; pain–hypercholesterolemia AOR = 0.447, 95% CI = 0.346, 0.577), good versus fair/poor perceived mental health status (pain–hypertension AOR = 1.534, 95% CI = 1.091, 2.157; pain–hypercholesterolemia AOR = 1.538, 95% CI = 1.079, 2.191), and having a functional limitation versus no functional limitation (pain–hypertension AOR = 1.442, 95% CI = 1.107, 1.879; pain–hypercholesterolemia AOR = 1.489, 95% CI = 1.105, 2.007).

Among the personal health practices, smokers with pain and hypertension were 1.5 times more likely to use opioids (AOR = 1.510, 95% CI = 1.131, 2.016), while smokers with pain and hypercholesterolemia were 1.6 times more likely to use opioids compared to non-smokers (AOR = 1.605, 95% CI = 1.207, 2.136). 

In the pain–hypertension group, those residing in the Northeast (AOR = 0.545, 95% CI = 0.385, 0.771) and Midwest (AOR = 0.675, 95% CI = 0.487, 0.935) were less likely to be opioid users compared to those residing in the West. Whereas, in the pain–hypercholesterolemia group, those residing in the Northeast (AOR = 0.593, 95% CI = 0.403, 0.872) were less likely to be opioid users compared to those residing in the West. 

None of the enabling factors were predictors of opioid use in either group. The fully adjusted binomial logistic regression models both had a Wald statistic of <0.0001 and a c-statistic of 0.700 (pain–hypertension) and 0.691 (pain–hypercholesterolemia).

## 4. Discussion

This retrospective, cross-sectional database analysis is the first to identify characteristics that were predictive of opioid use among these specific populations of older U.S. adults with pain and co-morbid hypertension or hypercholesterolemia. The need to understand predictors of opioid use among older adults is underscored by substantially increased use of opioid medications in this population in recent years. 

### 4.1. Predisposing Factors

Among the predisposing factors, race and ethnicity were associated with opioid use. Race (classified as “White” or “other”) was a predictor of opioid use in both the pain–hypertension and pain–hypercholesterolemia cohorts. This follows a long-established link between race and the likelihood of opioid analgesic use for pain management [33]. Ethnicity was associated with opioid use in both groups, with non-Hispanic respondents less likely to be opioid users when compared with Hispanic respondents, which matches a similar pattern shown in other research [33,34]. 

### 4.2. Need Factors

Among the need factors, our primary focus was chronic conditions. The list of chronic conditions was the same for both cohorts with the exception that the pain–hypertension group did not include hypertension and the pain–hypercholesterolemia group did not include hypercholesterolemia. The number of comorbidities was associated with increased likelihood of opioid use for both cohorts; having one additional comorbidity versus five or more was associated with a lower likelihood of opioid use in both groups, while having four comorbidities versus five or more was associated with increased likelihood of opioid use in the pain–hypercholesterolemia group. The reasons for association with these specific numbers of comorbid conditions are unclear, and further research is warranted to understand how the natural history of hypertension and hypercholesterolemia may impact the number and types of comorbidities and their attendant risk for opioid use. 

We also found perceived mental health status was associated with opioid use while perceived physical health status was not. Recent literature also reported an association between mental health disorders and opioid use in the U.S. [35,36]. There is also published evidence that depression symptoms can include physical pain and that depression can be linked to increased risk of developing comorbidities such as heart disease [37]. We therefore suggest that depression and other mental health conditions may be targets for intervention among older adults with pain to help reduce any unnecessary opioid use. 

Lastly among need factors, we found functional limitations (difficulties in performing activities such as lifting, walking, standing, bending, stooping, reaching, and grasping) were associated with increased likelihood of opioid use, yet limitations with ADL, IADL, or work were not. A previous study noted a relationship between depression, pain, functional limitations, and opioid use [38], which suggests several need factors may be related to an increased likelihood of opioid use and therefore warrants further investigation.

### 4.3. Personal Health Practice Factors

The personal health practice factor of smoking was associated with an increased likelihood of opioid use in both cohorts. Conversely, regular exercise was not associated with increased likelihood of opioid use for either cohort. Previous literature demonstrates that smokers are less likely to report regular physical activity [39]. Therefore, it was not surprising that reporting regular exercise was not associated with increased likelihood of opioid use, whereas reporting smoking was more associated with a higher likelihood of using opioids. 

### 4.4. External Environmental Factors

Region was the single external environmental factor included in our model, and we found those residing in the South were more likely to report opioid use than those in other regions (Northeast, Midwest, or West). Other literature has reported similar patterns of opioid use among U.S. adults [40,41]. Although the variations in regional use may be informative for policy development at the national level, there is large variation between states [42], thus we suggest more granular information is required for this information to be helpful at the state or local level for policy and planning initiatives. 

### 4.5. Prevalence of Opioid Use

In addition to predictors of opioid use, we also reported the extent of opioid use among these populations. We found that, among U.S. adults aged ≥50 years, 29.4% with pain and hypertension used an opioid and 28.5% with pain and hypercholesterolemia used an opioid. This parallels the findings of a recent CDC report that stated person-level prescribing of opioids increased with age, with 23.1% of adults aged 45–54 prescribed an opioid, 26.3% of adults aged 55–64 prescribed an opioid, and 26.8% of adults aged ≥65 years prescribed an opioid [42].

### 4.6. Implications for Clinical Care

Previous research that explored the multidomain strategies used to manage chronic pain found that non-steroidal anti-inflammatory drugs (NSAIDs) and opioids were the most commonly reported prescription pharmacological strategies [43]. One possible reason for the use of opioids among people with pain and hypertension is that some pain medications (e.g., NSAIDs) are contra-indicated for use with many hypertension medications [44,45]. Given the limited options for hypertension medications versus those for pain, perhaps clinicians prescribe opioids for pain management (especially acute use) if hypertension is well-controlled. However, there are fewer contraindications between NSAIDs and other pain medications and statins or other cholesterol medications [46,47], so a wider choice of pain management strategies are available. Non-pharmacological strategies such as exercise and relaxation are also commonly used for pain management [7], and may serve as alternative pain management strategies for older adults with pain and comorbid cardiovascular conditions.

### 4.7. Limitations and Future Work

Due to the nature of self-reported and secondary data, this study is subject to some limitations. First, this study could not determine a cause and effect relationship, although statistical associations could be determined. Second, although this study involved a nationally representative large sample, small intergroup differences could be statistically significant yet not clinically meaningful. Third, the narrowly defined groups (i.e., pain–hypertension, pain–hypercholesterolemia) limit the generalizability of the findings beyond individuals with these conditions. Fourth, recall bias from self-reported data may be present, although, to minimize this risk, MEPS interviews are conducted at regular intervals (every 4 to 5 months during the study period). Finally, we were unable to distinguish between acute and chronic opioid users, which would have added additional detail to our analyses. Although we focused on pain with the common cardiovascular conditions, hypertension and hypercholesterolemia, there are several other comorbid conditions of interest (e.g., diabetes, neurological conditions) that may be worthy of investigation to determine if similar patterns are also identified. Future research could also explore similar questions using a different dataset to determine whether the results can be replicated.

## 5. Conclusions

In this retrospective, cross-sectional database study, we found that approximately nine million U.S. older adults with pain and comorbid hypertension or hypercholesterolemia used opioids. Several characteristics were predictive of opioid use in both groups that may help target interventions to reduce inappropriate opioid use, but there were also differences between groups in some characteristics that warrant further investigation. 

## Figures and Tables

**Table 1:** Select characteristics of United States older adults (age ≥50 years) with self-reported pain in the past four weeks and a diagnosis of hypertension or hypercholesterolemia.

	Pain-Hypertension Group			Pain-Hypercholesterolemia Group		
Factors	Opioid User wt.% (95% CI)	Non-User wt.% (95% CI)	*p*	Opioid User wt.% (95% CI)	Non-User wt.% (95% CI)	*p*
**Predisposing factors:**						
Age ≥65 years	55.9 (52.0–59.8)	59.6 (56.5–62.7)	0.1279	56.6 (52.5–60.8)	60.6 (57.4–63.7)	0.1246
Non-Hispanic	94.5 (93.1–95.9)	89.9 (88.2–91.7)	<0.0001	94.7 (93.1–96.2)	89.5 (87.8–91.3)	<0.0001
White	81.1 (77.7–84.5)	76.5 (74.0–79.0)	0.0270	84.1 (81.1–87.1)	80.3 (78.0–82.5)	0.0383
Female	57.0 (53.1–61.0)	52.4 (49.8–55.0)	0.0456	53.7 (49.2–58.2)	52.6 (49.9–55.3)	0.6975
**Enabling factors:**						
>High school education	44.2 (39.3–49.1)	50.7 (47.6–53.8)	0.0161	45.0 (40.3–49.7)	50.5 (47.7–53.3)	0.0754
Unemployed	76.3 (72.3–80.3)	68.8 (65.7–72.0)	0.0030	73.6 (69.3–77.6)	67.7 (64.4–71.0)	0.0261
Married	49.8 (45.6–54.0)	56.0 (53.2–58.8)	0.0086	52.6 (47.9–57.3)	57.7 (55.0–60.4)	0.0401
Private health insurance	53.8 (49.4–58.1)	59.2 (56.3–62.2)	0.0581	55.9 (51.5–60.3)	60.1 (56.9–63.3)	0.1626
Middle/high income	59.7 (55.2–64.2)	65.6 (62.8–68.3)	0.0137	61.7 (57.0–66.5)	66.9 (64.2–69.7)	0.0348
**Need factors:**						
≥5 chronic conditions	41.0 (36.9–45.1)	31.2 (28.6–33.9)	<0.0001	39.6 (35.5–43.6)	32.0 (29.3–34.7)	<0.0001
Little/moderate pain	51.4 (47.3–55.5)	75.8 (73.3–78.3)	<0.0001	55.2 (50.9–59.4)	77.9 (75.4–80.4)	<0.0001
Ex/VG physical health	21.2 (17.8–24.5)	30.0 (27.4–32.7)	<0.0001	24.8 (21.3–28.2)	32.2 (29.7–34.7)	<0.0001
Ex/VG mental health	40.1 (35.9–44.3)	49.9 (46.9–52.9)	0.0004	41.9 (37.8–46.0)	50.4 (47.3–53.4)	0.0024
ADL limitation	9.9 (7.7–12.1)	5.8 (4.5–7.1)	0.0005	8.1 (6.1–10.0)	5.0 (3.8–6.2)	0.0070
IADL limitation	16.5 (13.5–19.4)	10.0 (8.4–11.6)	<0.0001	14.0 (11.2–16.9)	8.9 (7.3–10.5)	0.0011
Functional limitation	63.3 (59.3–67.3)	42.1 (39.6–44.7)	<0.0001	60.3 (56.1–64.5)	39.8 (37.2–42.5)	<0.0001
Work limitation	47.8 (43.5–52.1)	28.5 (26.1–30.9)	<0.0001	44.9 (40.6–49.1)	26.9 (24.3–29.4)	<0.0001
**Personal health practices:**						
Frequent exercise	32.5 (28.8–36.2)	37.8 (35.0–40.6)	0.0199	35.3 (31.5–39.2)	39.8 (37.2–42.5)	0.0459
Smoker	19.4 (16.2–22.6)	12.0 (10.5–13.6)	<0.0001	19.7 (16.6–22.7)	11.2 (9.7–12.7)	<0.0001
**External environmental:**						
South region	44.1 (39.5–48.6)	38.1 (35.0–41.3)	0.0096	43.3 (38.7–47.9)	37.6 (34.6–40.5)	0.0386

Pain–hypertension group: unweighted sample *n* = 2733 (opioid users *n* = 803; non-users *n* = 1930) reflects weighted sample *n* = 29,308,898 (opioid users *n* = 8,625,387; non-users *n* = 20,683,511) of United States adults alive during the calendar year 2017, age ≥50 years, with self-reported pain in the past four weeks, and a diagnosis of hypertension. Pain–hypercholesterolemia group: unweighted sample *n* = 2796 (opioid use *n* = 795; non-users *n* = 2001) reflects weighted sample *n* = 31,014,839 (opioid users *n* = 8,835,304; non-users *n* = 22,179,535) of United States adults alive during the calendar year 2017, age ≥50 years, with self-reported pain in the past four weeks, and a diagnosis of hypercholesterolemia. Statistically significant differences between groups based on chi-square tests. wt. = weighted; CI = confidence interval; Ex/VG = excellent/very good; ADL = activities of daily living; IADL = instrumental activities of daily living.
